# A scoping review of education and training resources supporting care home staff in facilitating residents’ sexuality, intimacy and relational needs

**DOI:** 10.1093/ageing/afab022

**Published:** 2021-03-03

**Authors:** Maria Horne, Jane Youell, Laura J E Brown, Paul Simpson, Tommy Dickinson, Christine Brown-Wilson

**Affiliations:** Faculty of Medicine and Health, School of Healthcare, University of Leeds, Leeds, UK; Faculty of Medicine and Health, School of Healthcare, University of Leeds, Leeds, UK; Division of Psychology and Mental Health, School of Health Sciences, University of Manchester, Manchester, UK; Department of Applied Health and Social Care, Edge Hill University, Ormskirk, UK; Department of Mental Health Nursing, Florence Nightingale Faculty of Nursing, Midwifery and Palliative Care, King’s College London, London, UK; School of Nursing and Midwifery, Queens University, Belfast, UK

**Keywords:** sexuality, older people, intimacy, training, education

## Abstract

**Background:**

Having positive intimate, sexual and relational experiences is an important issue for older adults in care settings, yet little is known on the extent to which nursing staff and care workers have received education or training in addressing and meeting these needs among older residents. This scoping review aimed to identify and examine what education and training resources exist to assist nursing staff and care workers to meet their residents’ needs in this area.

**Methods and analysis:**

Using the Arksey and O’Malley framework, we systematically searched papers and grey literature to identify education interventions and resources that aimed to facilitate care home staff to meet their residents’ sexuality, intimacy and relational needs.

**Results:**

Eleven studies (one dissertation) and three education resources met the inclusion criteria; most were conducted in the USA and Australia. Across the studies and resources identified, the education content was mixed and the methodology, presentation, design and duration varied widely. The focus of the education interventions and resources was to increase knowledge and improve and/or change attitudes towards the: (i) sexual expression of older people living in residential aged care, (ii) sexuality and ageing and (iii) expression of sexuality in people with dementia.

**Conclusion:**

Few education interventions and training resources were identified. The findings suggest that education interventions can improve knowledge and/or change care staff attitudes, in the short-term, towards older people’s sexuality, intimacy and relational needs in care home settings, which can lead to facilitating staff to enhance person-centred care in this area of need.

## Key points

- Identified what sexuality/intimacy education resources exist for care home staff
- Eleven education interventions identified from database searches, three education resources from systematic search of grey lit
- Education interventions can improve knowledge and/or change care staff attitudes towards older people’s sexuality/intimacy needs
- Future research needs to assess the medium- to long-term changes in attitudes, awareness and knowledge of care home staff
- Further research needs to measure the impact of changes in attitude and knowledge on care delivery and resident outcomes

## Introduction

Having positive intimate, sexual and relational experiences is an important issue for older adults. Sexuality and intimacy are an inherent and legal right [1] but remain a neglected area in promoting quality of life, well-being and personal identity [2, 3]. This is particularly the case for older adults residing in care settings, such as nursing and care homes [4, 5]. In the UK, over 400,000 older people reside in care homes [6]. Studies suggest that sexuality, intimacy and relationship needs still matter to many older adults in care settings [7] and remain an integral part of normal life for many older individuals. One study, drawing on the experiences of community dwelling older adults, found that 19% of men and 32% of women aged 80+ report having frequent sexual intercourse [8]. Intimate behaviours, such as frequent kissing and fondling, were reported by over half of respondents aged 80+ (men 47% and women 62%) [8], suggesting that sexual expression remains important in older age.

Such needs persist even when cognitive and physical decline advances to a stage where residential and nursing care is needed [9, 10]. When an older person moves into residential care, private lives are made more public with family members, staff and clinicians becoming increasingly involved in meeting care needs. This is particularly evident in those who have a diagnosis of dementia. The care environment is then perceived as a hindrance to managing intimate relationships. Staff report uncertainty in how to manage the many agendas at play from residents, family and staff, calling for more debate, discussion and training to question the views that residents are asexual and challenge the risk averse positionality in care provision [11]. Surveys suggest that intimate behaviours range from touching and kissing to masturbation and intercourse, which continue to remain important to long-term care residents [12]. However, stigma and stereotypical perceptions of sex and ageing persist [13]. One study sought the views of relatives and care workers of new relationships between residents with dementia whilst still married to another person. None of the care workers in this study referred to sources of advice or information [13], suggesting that staff were not aware of existing training resources in sexuality and intimacy or where to seek guidance. There remains a gap in knowledge in addressing and meeting the sexuality, intimacy and relational needs of older adults residing in residential care settings [14, 15].

Research highlights concerns about the ability of nursing staff in residential care settings to provide appropriate provision in this area of need and calls for a holistic approach and to move away from the biomedical and its emphasis on sexual function [15]. Additionally, there is limited research reporting on the extent to which nursing staff have received education or training in this area [4], although a review of knowledge and attitudes of healthcare professionals towards the sexuality of older people highlighted a general lack of knowledge and confidence in the area [16]. This is important as increased knowledge and awareness about the needs of care home residents in relation to sexuality, intimacy and relational needs has been shown to promote more positive and permissive nursing staff attitudes towards this area of person-centred care [17, 18].

Evidence suggests that the care home workforce are diverse, with differing educational levels, unclear career paths and varied experience in clinical settings [19]. This makes the development of effective education and training programmes challenging. In a review of dementia training literature relevance to practice, interactive group work with an emphasis on active learning with clear, easy to follow guidance were factors for effective training, which influenced practice [19]. This approach may be more problematic in sexuality, intimacy and relational training as there are ‘no right answers’, but consideration should be given on a case-by-case basis.

A cross-sectional postal survey of Australian residential aged care facilities found that only 45.6% of respondents reported that staff in their facility had attended training regarding later life sexuality and sexual health within the past 2 years, whilst 40.7% indicated that their staff had never received training in this area [4]. Most of the education and training was delivered by external educators (70.9%), with commonly covered topic areas including attitudes towards aged sexuality (70.0%), sexuality and normal ageing (69.9%), sexuality and dementia (68.7%), definition of sexuality (52.2%), disruptive sexual behaviour (46.3%), consent and legal issues (39.8%), sexual health (17.5%), guidelines/policy (16.3%), assessment (12.0%), sexuality and illness (10.8%) and pharmacological treatments (78%). The most cited ‘other’ subject area was sexual preference, specifically lesbian, gay, bisexual, transgender, intersex (LGBTI+) sexualities. We recognise that throughout this paper different abbreviations are used to represent the LGBTI+ community. This reflects the abbreviation used within the articles identified in the scoping review. More contemporary abbreviations reflect the broader LBGTI+ community, which is the authors’ preference, hence the variation in abbreviations.

Given the need for increased education and training to help staff to support care home residents’ sexuality, intimacy and relational needs, the purpose of this scoping review was to identify and examine what education and training programmes and resources (materials used for teaching a course) exist to facilitate care home staff to meet their residents’ sexuality, intimacy and relational needs.

## Methods

The scoping review follows the Preferred Reporting Items for Systematic Reviews and Meta-Analyses extension for scoping reviews (PRISMA-ScR) [20], employing the Arksey and O’Malley methodological framework for scoping reviews [21]. The sequential stages of this framework are to (i) identify the research question, (ii) identify the relevant literature, (iii) select the literature, (iv) chart the data and (v) collate, summarise and report results [22].

### Identifying the research question(s)

This scoping review was guided by the following questions:

- What existing education and training programmes and resources are there to facilitate care home staff in their support of older care home residents’ sexuality, intimacy and relational needs?
- What is the nature, extent and range of existing education and training programmes and resources to support care home staff to meet residents’ sexuality, intimacy and relational needs?
- What are the gaps in the evidence base?

### Identifying relevant literature

#### Search strategy

Searches were undertaken using five electronic databases: CINAHL, Embase, ERIC Medline, Scopus and ISI Web of Science, to capture a comprehensive sample of literature, for records published from 1980 to 30 March 2020 in the English language. This extended time period was chosen to identify relevant early education interventions. To ensure that all relevant information was captured, the grey literature was searched using google (two engines), three health and social care websites (National Institute of Clinical Excellence, Social Care Institute for Excellence and Public Health England), websites of relevant organisations (charities and organisations with an interest in aged care) and the grey literature databases (e.g. OpenGrey). Reference lists of included papers were also searched.

#### Search terms

Search terms were developed under the headings ‘homes for aged’, ‘Sexuality’, ‘Nursing’ and ‘Aged’ to ensure that all studies meeting the inclusion criteria were captured. Truncation (^*^) was employed where variations of a search term existed. A copy of the search terms used and modifications necessary across databases is available from the corresponding author upon request.

### Selecting the literature

Included sources from the published research were qualitative and quantitative studies that describe or evaluate any resource designed to help/train/educate care home staff to support/manage the expression of sexual or intimate behaviours; written in English and published from 1980. Included sources from the grey literature were theses/dissertations, reports and other sources that identified education and training programmes and/or resources to assist care home staff in their support of older care home residents’ sexual or intimacy needs. Excluded sources (from both the published research and grey literature) were those that (i) reported cases of sexual abuse in care homes and (ii) other systematic/scoping reviews.

### Charting the data

A data extraction sheet was developed to tabulate data related to study origin, study design, population characteristics, description of educational intervention/resource, outcomes measured and study results. This was undertaken by two authors (M.H. and J.Y.). Quality of study design was assessed using a validated checklist for evaluating studies of diverse designs [23]. Study eligibility was confirmed by two authors (M.H. and J.Y.). There were few initial disagreements related to inclusion or exclusion of the sources; decisions on inclusion/exclusion were achieved through consensus and did not require mitigation by a third author. Each paper was judged against 14-points on the checklist, where a single method was used, and 16-points on the checklist, where multiple methods were used. A percentage score was calculated as a measure of quality; a score of less than 50% was considered poor quality; a score between 50 and 70% was considered to be moderate quality and a score of greater than 70% was considered high quality. Results were synthesised narratively.

### Collating, summarising and reporting the literature

For study and training resources, a summary of the structural elements of the intervention as well as the content of the educational resource/training, outcome measures/learning outcomes and main findings were summarised and reported.

## Results

**Figure 1:**
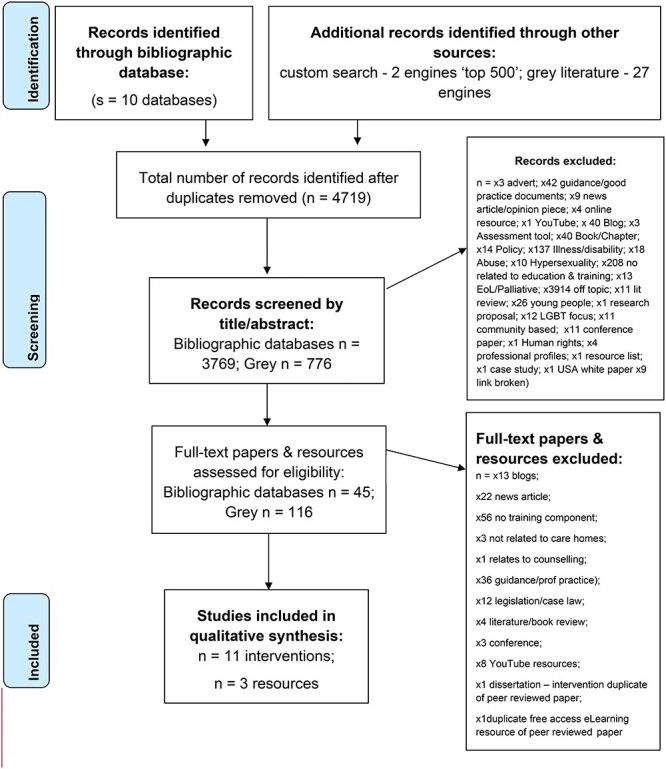
PRISMA flow diagram.

A total of 4,719 citations/resources were identified from searches of electronic databases and other sources (grey literature/Google), after duplicates were removed. Following the application of exclusion and inclusion criteria, 11 education intervention papers were identified [24–34]. In addition, the systematic search of the grey literature identified three education resources [35–37], which met the inclusion criteria (Figure 1).

Education intervention studies and resources were analysed for type of delivery, aims/learning outcomes of the educational content, outcomes measures, target audience (who it was aimed at), target population, study designs and broad findings (Tables 1 and 2). The study quality of the education interventions was poor to good (Table 1).

**Table 1:** Summary of relevant studies (a full table is available in Appendix 1 in Supplementary data are available in Age and Ageing online)

Study	Design	Intervention/duration	Participants and setting	Outcome measures	Main findings	Conclusion	Quality rating
**Aja and Self (1986) [** **24** **]** USA	RCT	• Two-day Sexual Attitude Reassessment (SAR) training programme—×14-h workshop split over 2 days. • Participant groups: **(i) Implicit group—e**xposure to sexually implicit materials; **(ii) Explicit group—e**xposure to sexually explicit materials; **(iii) Control group**	• *n* = 32 nurses’ aides, dietician aides, registered nurses, social workers and nursing home administrators from one nursing home.	• **Attitude and Knowledge:** (i) Sex, Knowledge and Attitude test (SKAT); (ii) LTK Attitude Rating Scale (Aja, 1982)	• Both treatment groups performed significantly better than the control group (*P* < 0.05) on the Knowledge Scale of the SKAT. • No significant differences based on analysis of the LTK Attitude Rating Scale.	• Changes in attitudes and knowledge can be achieved without the use of sexually explicit film materials.	13/42 31%
**Bauer *et al*. (2013) [** **25** **]** Australia	Pre-, post-test	• Education intervention specifically designed for nursing staff working in residential aged care delivered as a 3-h workshop • Participants completed a self-administered questionnaires at the beginning and at the end of the workshop.	• *n* = 112 (*n* = 102 female—93%) facilitated in community setting • Registered nurses and enrolled nurses/licensed practical nurses employed in two regional health services in Victoria, Australia.	• **Attitude:** (i) attitudinal part of the Ageing Sexuality Knowledge and Attitudes Scale (ASKAS) (ii) 8/20 items from the Staff Attitudes about Intimacy and Dementia (SAID) Survey (Kuhn, 2002).	• Attitudes towards sexual expression of older people in long-term care were significantly more permissive following the education intervention • No significant differences between pre- and post-testing based on demographic variables—gender, age, English as a first language, job position, years worked in aged care or study site.	• Short duration education intervention can have a significant impact on the permissiveness of staff attitudes, including same sex relationships and people with dementia, towards sexuality of older adults living in residential aged care and older adults with and without dementia.	29/42 59%
**Hammond and Bonney (1985) [** **26** **]** USA	Pre-, post-test	• Sexuality and ageing course; consisted of a 7-week course, meeting for 2 h/week each week (total 14 h) using a blended learning. • Experimental group attended course; control group did not. • Both experimental and control groups completed pre-test and post-test surveys.	**Experimental group:** *n* = 28 participants **Control group:** *n* = 25 participants	• **Attitude and Knowledge:** Attitudes towards sexuality in the aged: community aged tool (White, 1978)	• Significant increase in knowledge scores about sexual ageing was seen in the experimental group (*F*_1,51_ = 36.9340; *P* = 0.0001) compared to the control group. • Significant change in attitude scores was seen in the experimental group compared to the control group (*F*_1,51_ = 6.0632; *P* = 0.0172).	• Experimental group demonstrated more liberal viewpoints in attitudes compared to the control group (no change in attitudes). • Evidence of change towards a more liberal attitude and confidence in practice.	17/48 35%
**Jones and Moyle (2016) [** **27** **]** Australia	MM	• Intervention based on the sexualities and dementia: Education resource for health professionals (Jones and Moyle, 2014) using a blended learning approach. • Prior to and upon completion of the eLearning education resource, participants completed a pre- and post-intervention online questionnaire. • Subset of participants took part in semi-structured interviews.	*n* = 16 nursing students and *n* = 26 registered nurses, enrolled nurses, personal care workers and therapists working in RACFs.	• **Attitude and Knowledge:** (i) The Ageing Sexual Knowledge and Attitudes Scale (ASKAS). (ii) The Staff Attitudes about Intimacy and Dementia (SAID) Survey. (iii) Interviews	• Statistically significant change between participants’ pre- and post-ASKAS knowledge scores (*Z* = −2.82, *P* = 0.005) with lower ASKAS knowledge items scoring lower in the post-test (M = 51.0; SD = 8.56) than pre-test (M = 57.57; SD = 15.06) indicating increased knowledge • Significant differences were found for both ASKAS (Z = −2.57, *P* = 0.01) and SAID (Z = −3.14, *P* = 0.002) attitude scores indicating improved attitude and permissiveness.	• Staff knowledge was significantly improved and attitudes were significantly more permissive towards the expression of sexuality by people with dementia living in RACFs following completion of the self-directed eLearning education intervention.	32/48 67%
**Livni (1994) [** **28** **]** South Africa	QE	• Educational programme, delivered as a 62-min video-guided, facilitated discussion workshop. • Pre-test and post-test questionnaires undertaken • **Control group:** Only completed the questionnaire.	• Pre-test *n* = 210 nursing staff: (i) experimental group *n* = 120; (ii) control group *n* = 90. • Post-test *n* = 183 nursing staff: (i) experimental group *n* = 101; (ii) control group *n* = 82.	• **Attitude and Knowledge** (i) Ageing and Sexuality Knowledge (ii) Attitude Scale for Dementia (DEMASKAS).	• **DEMASKAS:** Nurses knowledge improved significantly with regard to sexuality and dementia in the experimental group. • **Repeated measures MANOVAS:** significant differences were found from pre- to post-test on (i) general morality (ii) institutional sexuality (iii) dementia and sexuality (experimental group)	• Attitudes changed significantly in a more tolerant direction after an educational program, except towards patients with severe dementia. • No significant correlation seen between attitude change and demographic variables or prior knowledge of sexuality in dementia.	24/42 57%
**Mayers and McBride (1998) [** **29** **]** USA	Pilot study	• Pilot training programme delivered as a ×3-h workshop: • Delivered as group activities, open discussions and handouts to consolidate learning	• *n* **=** 27 including psychiatrists, physicians, nurses and nurse administrators, social workers, therapists and students. • 20 participants undertook a post-training interview	• **Efficacy of the training:** standard hospital feedback form. • **Attitude and Knowledge:** (i) sexual attitude survey (ii) individualised interview 5 months post-training	Only qualitative statements. • Need for education to ensure staff are reflective of own biases and misconceptions. • Training enabled staff to consider ageing and dementia and their attitudes towards older age sexuality.	• The training program on sexuality was quite effective in eliciting interest and participation. • Methods used to enable staff to discuss the topic openly were well generally accepted.	13/48 27%
**Menzel (2005) [** **30** **]** USA	QE	• Training programme consisted of ×1-h face-to-face presentation, supplemental handouts, video footage and in group discussion with case studies/case vignettes: • **Experimental group**: received training program. Pre- and post-test measures were undertaken • **Control group** (a second nursing home): completed pre- and post-test measures at the same time as the experimental group.	• *n* = 35 nursing home staff in western Pennsylvania: (i) Experimental group *n* = 29 undertook training (ii) Control group *n* = 6 • *n* = 20 completed a questionnaire and take part in a short interview pre- and post-test	• **Knowledge:** researcher-developed questionnaire. • **Attitude:** researcher-developed questionnaire. • **Incidence of sexual behaviour in the past month:** interview questions.	• Significant increase in the number of reported sexual behaviours from pre- to post-test. • Significant differences were found in attitudes towards geriatric sexuality from pre- to post-test. • Although no significant increase in knowledge of geriatric sexuality were found, the trend suggested that staff training had a positive impact.	• Significant decrease in reported incidence of sexual behaviour at post-test in experimental group; suggests that staff training had an impact on the reporting of these behaviours. • Training programme had a greater impact than anticipated in relation to discussions about policy in nursing homes.	N/A—dissertation
**Reingold and Burros (2004) [** **31** **]** USA	Unclear	• Policy and video training program on sexual expression; duration not specified.	• Nurses, aides, physicians, social workers, dieticians and activity co-ordinators from one nursing home with 17 units.	• None	Only qualitative statements provided. • Staff education was received positively. • Staff were relieved to have clearer guidelines on how to respond to situations that had made them anxious, uncertain, and uneasy.	No outcome measures utilised. No specific demographic details reported.	5/42 12%
**Steinke (1997) [** **32** **]** USA	Pilot study	• Education intervention on sexuality in ageing, delivered as two one-half day (1 week apart) sessions. • Participants completed: (i) pre-test demographic tool and three general questions related to sexuality and privacy issues; (ii) pre- and post-test ASKAS.	• *n* = 10 (Female = 8; Male = 2) registered nurses, licensed practical nurses, certified medication assistants, certified nursing assistants	• **Attitude and Knowledge:** ASKAS. • **Sexuality and privacy issues**: assessed through three general questions.	• Significant increases seen in knowledge about sexuality from pre-test to post- test; t (9) = 4.27, *P* = 0.002. • No significant differences seen in attitude scores from pre- to post-test; *t* (9) = 0.72, *P* = 0 0.49	• Educational intervention can increase knowledge about sexuality in ageing. • No change in attitudes observed.	15/42 36%
**Walker and Harrington (2002) [** **33** **]** USA	E	• Sex and Sexuality in Long-Term Care curriculum delivered over a 3-week period. • Included long-term care facilities were not offered the same modules, therefore duration varied. Participants attended one or more training sessions.	• Convenience sample of *n* = 109 (*n* = 99 female; *n* = 10 male), long-term care staff from four settings who completed one or more of the four modules.	• **Attitude and Knowledge:** Knowledge and Attitudes Toward Elderly Sexuality (KATES)	• **Training effect on knowledge and attitudes towards older people sexuality:** Main effect of time (*P* < 0.0005) was significant, indicating that post-test scores were higher than pre-test scores.	• Training modules were successful at improving long-term care staff knowledge of and attitudes towards older people sexuality. • Improvement was not uniform across modules.	32/42 76%
**White and Catania (1983) [** **34** **]** USA	RCT	• A sexual psychoeducational intervention consisting of panel discussions • **Duration:** (i) Older adults: ×3, 2-h sessions; (ii) family of older adults: 1-day, 6-h session; (iii) nursing home staff: 1-day, 6-h session • **Control group**: pre- and post-test measures via personal interview.	• **Nursing home staff participants:** (i) Intervention *n* = 30; (ii) controls = *n* = 33. • **Older adults:***n* = 30 (12 men; 18 women) community dwelling older adults; • **Family of older adults:** *n* = 30 close family member.	• **Attitude and Knowledge:** Assessed using ASKAS scale • **Sexual behaviour questionnaire** for older adults only	• Significant changes in knowledge about and attitudes towards sexuality and ageing in all three experimental cohorts post-intervention, exposure to programme had significant positive effect in all groups. • Significant increase in recognising the importance of sexuality. • Significant increase (400%) in sexual behaviour in experimental group.	• Significant changes in attitudes toward and knowledge about sexuality and ageing and sexual behaviour.	24/42 57%

RCT = randomised control trial, MM = mixed methods, QE = quasi experimental, E = evaluation

**Table 2:** Education resources related to sexuality, intimacy and relational needs of older care home residents

Resource name	Content	Target audience	Target populations	Learning outcomes
**Alzheimer’s Society—Lift the Lid (2019) [** **35** **]** Pay for resource UK	• Pay for workshop in a box on sex, intimacy and relationships developed by Alzheimer’s Society’s Innovation team. • **Box Contains:** Lift the Lid leaflet and a facilitator guide and three activities: (i) A true or false game—10 question and answer cards to challenge perceptions/provoke discussion. (ii) Follow the heart—uses scenarios and guidance to support important conversations with residents, partners and families. (iii) Plan for Change—to help staff identify what will make a practical difference in their care home.	Care home staff	People with dementia living in care homes	• Not specifically reported. • Designed to challenge perceptions around sex, intimate relationships and people affected by dementia and provide a framework for respectful management of in-the-moment situations, make practical changes, e.g. reviews of residents’ care plans to ensure that emotional and psychological well-being are included; assist care home staff to create their own policies based on individual need and consent. • Audience-led innovative sprints to gather knowledge, actionable insights and creative solutions to prototype, adapt and adopt. The shared outcomes and process are intended to be used to help the care home meet the needs of people living with dementia.
**Aged Care Awareness (2020) [** **36** **]** Free access resource. Australian	• Aims to showcase the increasing complexity of aged care; challenge attitudes and assumptions; develop understanding of how quality care can be delivered to older people. • Four modules in total of which module 2 is relevant to sexuality, intimacy and relationship needs: • Module 2: focuses on Resident’s rights and intimacy and aims to increase awareness and understanding of the complexity of practice in supporting resident autonomy and personhood. • Contains four short films that explore the relationship between two residents.	Health professionals in aged care.	Older adults in residential care People with dementia	**Module 2:** Through reflection of the films you will be able to: • Demonstrate an understanding of resident rights within residential aged care • Reflect on the complexity of supporting the rights of older people and the way policy can influence practice • Analyse your attitudes towards sexuality and sexual expression in older people • Identify additional resources to enhance your learning
**Sexuality, Intimacy and Dementia in Residential Care Settings (David Khun)** **Producer: Terra Nova Films, Inc. (2017) [** **37** **]** Pay for resource Israel	• A pay for 5-chaptered DVD designed to explore sexuality, intimacy and dementia, as well as the complex issues that impact residents, family members and care staff. • Consists of five 15- to 20-min videos: (i) The Effects of Dementia on Intimacy and Sexuality; (ii) Responding to Sexual Expressions;(iii) Consensual Intimacy and Sexuality; (iv) Spousal and Family; Responses; (v) Non-consensual Intimacy and Sexuality • The pack includes a PDF facilitator’s guide with key lesson points, discussion questions, sample policy and procedures with regard to sexual expression.	Care home staff	People with dementia residing in residential care	• To equip care staff with a well-rounded understanding of the sensitive issues concerning intimacy, sexuality and the rights of persons with dementia, as well as, how to respond to expressions of sexuality in a manner that promotes both resident dignity and safety. • DVDs explore issues of intimacy and sexuality on quality of life, freedom to express sexuality, capacity to consent, resident protections and potential legal ramifications. • Touches on the needs of lesbian, gay, bisexual, transgender (LGBT) residents, how to address resident-to-resident and resident-to-visitor encounters, and how to find workable solutions with the support of family members.

Eight education intervention studies were conducted in the USA [24, 26, 29–34], two in Australia [25, 27] and one in South Africa [28]. Of the three education resources identified through the grey literature/Google search, one pay for workshop was developed and available to purchase in the UK [35]; one free online resource was developed in Australia [36] and one-pay-for-view DVD resource and facilitators guide was developed in Israel [37].

## Description of education interventions and resources

### Content coverage

The education content was mixed across education interventions and resources; Tables 3 and 4 summarise the content. Defining sexuality/sexual behaviour/facts about sex and ageing was a common theme across all the education interventions and resources. Six of the 11 education interventions [25, 26, 29–32] focused on sex and sexuality as opposed to intimacy and relational needs; all the education resources [35–37] focussed on sexuality, intimacy and relational needs (Table 4). Dementia and challenging perceptions around sex and intimate relationships of people affected by dementia, featured often in the education interventions [25–28, 31, 33] and resources [35, 37].

**Table 3:** Summary of education interventions taught content

General subject/topic areas (identified directly from the resources)	Study										
	Aja and Self (1986) [24]	Bauer *et al*. (2013) [25]	Hammond and Bonney (1985) [26]	Jones and Moyle (2016) [27]	Livni (1994) [28]	Mayers and McBride (1998) [29]	Menzel (2005) [30]	Reingold and Burros (2004) [31]	Steinke (1997) [32]	Walker and Harrington (2002) [33]	White and Catania (1983) [34]
What is sexuality and its importance		X					X				X
Terminology, definitions, myths and facts related to sexuality and ageing			X			X			X		X
Intimacy, sexuality and sexual behaviour	X			X	X					X	X
Attitudes towards sexuality in older people		X				X	X		X		
Sexual-related stereotypes	X	X					X		X		
Non-heterosexuality-related stereotypes	X	X									
Physiological aspects of sexuality and ageing		X					X		X	X	X
Psychological aspects of sexuality and ageing									X		X
Sexuality and cultural differences			X								
Sexuality, illness and treatment		X	X				X		X		X
Disability and sexuality	X		X								
Sexual expression in residential care	X	X									
Expression of sexuality and people with dementia		X		X	X			X		X	
Sexuality and communication	X		X			X					
Specific sex acts	X				X		X				
Family and personal issues										X	
Approaches to sexuality and the role/responsibilities of residential aged care staff		X							X		
Sexuality and residents’ rights		X				X		X			
Legal issues related to sexuality		X									
Ethical considerations				X				X			
Development of sexualities and dementia policy guidelines for care practice/settings				X				X			

Most of the education interventions focused on facts around the physiological aspects of sexuality and ageing (Table 3). Two education interventions included non-heterosexuality-related stereotypes [24, 25], whilst one education resource touched on the specific needs of LGBTI residents [37], one education intervention included legal issues [25], cultural difference was only addressed in one education intervention [26] and family and personal issues was only mentioned in one education intervention [33].

Three education interventions [25, 29, 31] and two education resources [36, 37] explicitly framed sexuality as a rights-based and legal issue, whilst two education interventions [27, 31] and one education resource [36] specifically identified ethical issues in this area. Family and personal issues were only mentioned in one education intervention [33].

Communication was addressed in three of the education interventions [24, 26, 29] and two education resources [35, 37]. Only two education interventions looked at what organisations could do more broadly to support sexual expression [27, 31]. Specific sex acts were addressed in three education interventions [24, 28, 30].

### Methodology, presentation and design

Ten of the eleven education interventions [24, 25, 26, 28–34] and one pay for education resource [35] used active learning strategies, delivered in a workshop format. Each workshop used a range of methodologies such as presentations, vignettes, facilitated discussions, watching of video/film clips with facilitated group discussions, to explore ideas and develop knowledge. The use of written hand-outs helped to facilitate a better understanding of older people’s sexuality. One education intervention [27] and one education resource were delivered via an eLearning platform [36]; a final resource was a pay-for-view DVD set and a facilitator’s guide with key lesson points and discussion questions related to sexual expression, sexuality, intimacy and dementia [37].

None of the education interventions or resources explicitly articulated the use of educational theory to guide the design of the courses/resources.

### Duration

The education interventions and resources identified were quite varied in their duration of delivery taking the form of a 1-h PowerPoint training program [30]; a 62-min video-guided, facilitated discussion workshop [27]; a one off 3-h workshop [25, 29]; a ×14-h workshop over 2 days [24]; a two, one-half day (1 week apart) education session [32]; a 1-day, 6-h session [34]; a four 60-min programme topics over a 3-week period [33]; an up to 4-week completion of a self-directed, eLearning educational resource [27]; a 7-week face-to-face programme, meeting for 2 h/week, each week for a total of 14 h [26] and the duration was not specified in one educational intervention (Table 1) [31].

### Aims of education interventions and resources

The focus of the education interventions were to increase knowledge and improve and/or change attitudes towards the (i) sexual expression of older people living in residential aged care [25, 26, 31, 33]; (ii) sexuality of older people/sexuality and ageing [30, 32, 34]; (iii) expression of sexuality by people with dementia/differing stages of dementia residing in care settings for older adults [27, 28] and (iv) need for consideration of sexuality in geriatric programmes [24, 29].

The overall aim of the education interventions and resources was centred on what care staff could do to support older adults in their care in this area of need and little about organisational attitude towards supporting the needs of older adults except for one education resource [35].

### Staff attitudes, knowledge and understanding

Eight of the 11 education intervention studies used standardised psychometric measures to assess participant attitudes, knowledge and understanding pre- and post-educational intervention: (i) Ageing Sexuality Knowledge and Attitudes Scale [25, 27, 32, 34]; (ii) Staff Attitudes about Intimacy and Dementia Survey [25, 27]; (iii) the Ageing and Sexuality Knowledge and Attitude Scale for Dementia [28]; (iv) the Knowledge and Attitudes Toward Elderly Sexuality, developed by the team in previous research [33]; (v) the attitudes towards sexuality in the aged: community aged tool developed by White (1978) [26] and (vi) Sex, Knowledge and Attitude test [24].

One education intervention did not assess staff attitude via a recognised scale but through workshop discussions and a post-training interview [29]. One further study assessed participant attitude via non-validated, researcher-developed questionnaires [30]. One education intervention did not assess staff attitudes as part of their study [32].

Six education interventions reported statistically significant difference in scores towards more permissive attitudes regarding sexuality following the educational interventions [25–28, 30, 34] and one reported more positive changes in attitude towards sexual behaviour of older adults [29].

One education intervention assessed knowledge through a researcher-developed questionnaire [30]. A further study did not assess knowledge via a recognised scale, but through workshop discussions and a post-training interview [29]. Staff knowledge and understanding was not assessed in two education interventions: (i) the focus of the intervention was on permissiveness of staff attitudes towards sexuality of older adults with and without dementia [25] and (ii) staff knowledge and understanding were not assessed as part of the study [31].

Seven education interventions reported a change in knowledge relating to older people’s sexuality following the education intervention [24, 25–28, 32, 34].

**Table 4:** Summary of education resources content

General subject/topic areas (identified directly from the resources)	Education resource		
	Lift the lid	Aged care awareness	Sexuality, intimacy and dementia in residential care settings
Importance of expression of sexuality for people with dementia			X
Myths and facts related to sexuality and ageing	X		
Intimate and sexual needs of people with dementia			X
Attitudes towards sexuality and sexual expression in older people		X	
Attitudes/perceptions around sex, intimate relationships and people affected by dementia	X		
Supporting the expression of sexuality by people with dementia			
How to respond to expressions of sexuality that promotes dignity and safety for residents with dementia	X		X
Needs of LGBT residents			X
Communication related to sex and intimate relationships with residents, partners and families	X		X
Assessment of cognitive competency of people with dementia to have intimate and sexual relationships/capacity to consent			X
Approaches and the role/responsibilities of healthcare professionals/aged care staff in responding to the expression of sexuality by people with dementia	X		
Addressing resident-to-resident and resident-to-visitor encounters			X
Sexuality and residents’ rights and supporting the rights of older people		X	
Legal issues related to sexuality			X
Knowledge translation of sexualities and dementia into care practices/settings	X		
How policy can influence practice in supporting the rights of older people		X	

## Discussion

To the best of our knowledge, this scoping review is the first to identify what education and training resources are available to care home staff to assist them in meeting their residents’ needs in this area. This is important given that there remains a gap in knowledge in addressing and meeting the sexuality, intimacy and relational needs of older adults residing in residential care settings [14, 15].

The scoping review has identified a number of education interventions and resources aimed at improving awareness, knowledge and understanding about the sexuality and/or intimacy needs of older care home residents and to facilitate care staff to support older care home residents’ sex and sexuality needs, but few focussed on intimacy and relational needs or explicitly framed sexuality as a rights-based issue for older adults. Evidence suggests that intimacy is important for relational behaviour in care home settings to reduce loneliness and isolation [38] and therefore is an important consideration when delivering rights based, person-centred care.

The nature, extent and range of existing education interventions and resources to support care home staff to meet residents’ sexuality, intimacy and relational needs were varied in their content and delivery; dementia focussed in the main, but very few interventions and resources considered the specific needs of the LGBTQI+ residents. This is an important area of consideration for residential care homes around inclusivity and acknowledging the specific needs of this group of older adults. It should be noted that the scoping review contained papers from the USA, Australia, South Africa, the UK and Israel. The differing organisational cultures of residential care in these locations may have implications for education and training.

This scoping review has also identified that educational interventions have the potential to change care home staff attitudes towards older people’s sexuality and intimacy needs in a more permissive direction, supporting previous research in the area that suggested an increase in knowledge about sexuality was linked with more permissive and open attitudes towards sexual expression in older people [39].

Most of the education interventions and resources appear to move beyond simple provision of information and guidance with active learning focussing on developing “common sense” approaches and taking the resident’s perspective. Definitions of common sense vary across theoretical frameworks [40]. Common sense implies a shared set of values and beliefs, which guide decisions and practice [41]. However, sexuality and intimacy for older residents is subject to beliefs of ageist erotophobia, which necessitates the need to tackle biases of ageism, heteronormativity and asexuality in ageing [15], thus calling into question the validity of a “common-sense” approach.

Few education interventions and resources specifically looked at communication around how to manage sexuality, intimacy and relational needs with residents, partners and family. Previous reviews have identified that this is an area where care home staff identify that they need further guidance [42]. In addition, what organisations could do more broadly to support sexual expression, intimacy and relational needs was lacking. A supportive culture in the organisation has been identified as an important facilitator to improving care staff attitudes towards resident sexuality [43].

The influence of culture and ethnicity on sexuality [44] was explored in only one education intervention [26]. The impact of organisational culture on the attitudes of residential care staff towards the sexuality and intimacy of care home residents [43] was not explored in any of the education interventions and resources identified.

This scoping review identified and described the nature, extent and state of education and training resources undertaken to assist care home staff in managing older care home residents’ sexuality, intimacy and relational needs. This scoping review did not include an optional final step involving key stakeholder consultation, practitioners and policy makers [21]; this may have provided some additional insight into education and training resources. Although a rigorous approach to conducting the scoping review of the peer-reviewed and grey literature was undertaken using a recognised framework, it is possible that not all relevant records were identified in this area.

## Conclusions

Few education interventions and training resources were identified in this review. The education interventions did show potential in improving knowledge and/or changing nursing staff attitudes, in the short-term, towards older people’s sexuality, intimacy and relational needs in care home settings. All the papers identified in the scoping review highlight the importance of training in sexuality and intimacy. This can lead to facilitating staff to enhance person-centred care in this area of need for older adults living with and without dementia. However, the overall quality of five of these interventions was poor; therefore, the findings need to be interpreted with caution.

Future research is needed using prospective longitudinal approaches to assess the medium- to long-term changes in attitudes, awareness and knowledge of care home staff and the impact of changes in attitude and knowledge on care delivery and resident outcomes. In addition, further development of education and training resources that appeal to a diverse workforce and are situated in practice are needed.

## Supplementary Material

aa-20-1440-File002_afab022
