# Supplementary material for: A scoping review of education and training resources supporting care home staff in facilitating residents’ sexuality, intimacy and relational needs
Source: Age Ageing. 2021 Mar 3;50(3):758–71. doi: 10.1093/ageing/afab022 (PMC8123381; doi:10.1093/ageing/afab022)
Supplement: aa-20-1440-File002_afab022 [file aa-20-1440-file002_afab022.docx]

A scoping review of education and training resources supporting care home staff in facilitating residents’ sexuality, intimacy and relational needs.

**SUPPLEMENARTY DATA**

**Appendix 1: Educational interventions related to sexuality, intimacy and relational needs of older care home residents**

| **Study** | **Design & aim** | **Intervention/duration** | **Content** | **Participants & setting** | **Outcome measures** | **Main findings** | **Conclusion** | **Quality rating** |
| --- | --- | --- | --- | --- | --- | --- | --- | --- |
| **Aja & Self (1986) [20]**  USA | Randomized control study.  To determine if attitudes and knowledge of care home staff change when exposed to different levels of explicitness in sex related materials. | Two-day Sexual Attitude Reassessment (SAR) training programme consisted of a x14-hour workshop split over 2 days.  Three participant groups:  **(i) Implicit group**  **(ii) Explicit group**  **(iii) Control group** | **(i) Implicit group:**  Exposure to sexually implicit materials.  **(ii) Explicit group:**  Exposure to sexually explicit materials.  Both groups also completed exercises on values, sexual language & touching. | n=32 nurses’ aides,  dietician aides,  registered nurses, social workers & nursing home administrators from one nursing home. | **Attitude &**  **Knowledge**  Assessed by using:  (i) Sex, Knowledge & Attitude test (SKAT);  (ii) LTK Attitude Rating Scale (Aja, 1982) | Both implicit and  explicit treatment groups performed significantly better than the control group (p< .05) on the Knowledge Scale of the SKAT.  No significant  differences based on analysis of the LTK Attitude Rating Scale. | Changes in attitudes & knowledge can be achieved without the use of sexually explicit film materials. | 13/42  31% |
| **Bauer et al. (2013) [21]**  Australia | Pre-post-test questionnaire design.  To evaluate an education intervention designed for aged care nursing staff to increase knowledge & improve attitudes towards the sexual expression of older people living in residential aged care | Education intervention specifically designed for nursing staff working in residential aged care.  Delivered as a three-hour workshop.  Participants completed a self-administered questionnaire at the beginning & at the end of the workshop. | **Topics covered in the workshop:**  (i) importance of sexuality  (ii) common attitudes/stereotypes  (iii) sexuality & ageing  (iv) sexual expression in residential care.  (v) sexuality & people with dementia; (vi) residents’ rights and staff responsibilities.  (vii) legal issues including capacity & consent. | n= 112 ( n=102 female – 93%) facilitated in community setting    Registered nurses & enrolled nurses/licensed practical nurses employed in two regional health services in Victoria, Australia. | **Attitude** Assessed by using:  (i) attitudinal part of the Ageing Sexuality Knowledge & Attitudes Scale (ASKAS)administered pre and post workshop  (ii) 8/20 items from the Staff Attitudes about Intimacy and Dementia (SAID) Survey (Kuhn, 2002). | Attitudes were significantly more permissive following  the education intervention across both ASKAS attitudinal items (X^2^ = 11.5, df = 2, p<.01, n = 218) & the additional items from the SAID survey (X2 = 10.5, df = 2, p<.01,  n = 218).  No significant differences between pre- and post-testing based on demographic variables - gender, age, English as a first language, job position, years worked in aged care; or study site. | A relatively short duration education intervention can have a significant impact on the permissiveness of staff attitudes towards sexuality of older adults living in residential aged care & older adults with & without dementia.  Attitudes improved most for ASKAS items relating to ‘‘staff understanding of sexual needs’’ & for the dementia-specific items from the SAID survey.  Additional items from the SAID survey relating to homosexual relationships was also found to elicit a more permissive attitude following the education intervention. | 29/42  59% |
| **Hammond & Bonney (1985) [22]**  USA | Pre-test, post-test, evaluated with a non-randomised control group.  To investigate the effects of a course on sexuality & ageing on the acquisition of knowledge & attitude change. | Sexuality & ageing course; consisted of a 7-week course, meeting for two hours per week each week (total 14 hours) using a blended learning presentation.  Experimental and control group. Experimental group attended course, control group did not. Both experimental and control groups completed pre-test and post-test surveys. | Course objectives:  (i) Increased knowledge of sexual terms & functions  (ii) Understanding of own sexual biases  (iii) Distinguishing myths from facts  (iv) Effect of cultural differences  (v) Identifying developmental stages & psychological changes  (vi) Problems of the institutionalised  (vii) Implications for counselling | **Experimental group:** n = 28 participants enrolled in a gerontology certificate programme  **Control group:**  n = 25 participants enrolled in a gerontology certificate programme | **Attitude &**  **Knowledge:**  Assessed using the Attitudes toward sexuality in the aged: community aged tool (White, 1978) | A significant increase in knowledge scores about sexual aging was seen in the experimental group (F1,51 = 36.9340; p = 0.0001) compared to the control group  A significant change in attitude scores was seen in the experimental group compared to the control group (F1, 51 = 6.0632; p = 0.0172). | Experimental group demonstrated more liberal viewpoints in attitudes compared to the control group (no change in attitudes).  A fourteen-hour course can increase knowledge significantly.  Evidence of change toward a more liberal attitude, with the majority of course participants feeling more able to deal openly with the elderly in helping them with sexual problems. | 17/48  35% |
| **Jones & Moyle (2016) [23]**  Australia | Sequential mixed methods study using (i) online self-directed eLearning  training & online questionnaire (ii) subsequent semi-structured  interviews.  To evaluate the ease of use, quality, and effectiveness of an eLearning  education intervention to increase knowledge and improve attitudes of staff toward the expression of  sexuality by people with dementia living in Residential Aged Care Facilities (RACFs). | Participants had up to 4 weeks to complete the eLearning educational resource.  The education intervention was based on the Sexualities and dementia: Education resource for health professionals (Jones & Moyle, 2014) developed for the Dementia Training and Study Centre (Queensland-DTSC).  Upon completion of the eLearning education resource, participants completed the post intervention online questionnaire.  Subset of participants were interviewed:  (i) presented with two case scenarios of intimate and/or sexual relationships involving an older person with dementia; participant views of, and their approach and response to, the scenarios were sought and  (ii) evacuated the ease of use, quality & effectiveness of the eLearning education resource. | Consisted of four learning modules:  (a) intimacy, sexuality & sexual behaviour;  (b) dementia & the expression of sexuality; (c) ethical considerations: policy guidelines  development for sexualities & dementia in care settings;  (d) developing sexualities & dementia policy guidelines for care practice.  Case studies, activities, & resources are provided to facilitate & consolidate learning about the various content focus areas. | n = 16 undergraduate nursing students and n = 26 registered nurses, enrolled nurses, personal care workers & diversional therapists working in RACFs.  Prior to completion of the  education intervention, participants completed an online questionnaire that consisted of questions seeking  demographic information as well as their knowledge and attitudes toward the expression of sexuality by  older people in RACFs. | **ELearning resource:**  Knowledge & attitudes of participants toward the expression of sexuality by older people in RACFs were assessed using:  (i) The Aging Sexual Knowledge & Attitudes Scale (ASKAS).  (ii) The Staff Attitudes about Intimacy & Dementia (SAID) Survey.  **Interviews:** Views of & their approach & response to two case scenarios (their decision-making process & what they thought). | **Knowledge of late life sexuality:**  Statistically significant change between participants’ pre- & post-ASKAS knowledge scores (Z = −2.82, p = .005) with lower ASKAS knowledge items scoring lower in the post-test (M = 51.0; SD = 8.56) than pre-test (M = 57.57; SD = 15.06).  **Attitudes toward late life sexuality:**  Significant differences were found for both ASKAS (Z = −2.57, p = .01) and SAID (Z = −3.14, p = .002) attitudes scores. The total score  for the ASKAS attitude items at post-test (M = 41.10; SD = 11.97) was lower than at pre-test  (M = 48.76; SD = 16.51).  Total score for the SAID attitude items at post-test (M = 37.38; SD = 7.48) was lower than at pre-test (M = 41.90; SD = 10.88). | Staff knowledge was significantly improved & attitudes were significantly more permissive toward the expression of sexuality by people with dementia living in RACFs following completion of the self-directed eLearning education intervention.  Staff appeared to be less reliant on cognitive capacity assessment, focusing on the overall happiness and well-being of residents & strategies to respond to intimate and/or sexual relationships of people with dementia.  Mode of education delivery lacks the ability to engage in discussion, interaction & support for change in practice.  Although the education intervention was effective in improving knowledge & nurturing more permissive attitudes in staff, to what extent (if any) this change impacted on practice was not examined. | 32/48  67% |
| **Livni (1994) [24]**  South Africa | Quasi-experimental design.  To examine the knowledge of and attitudes toward sexuality and differing stages of dementia | Educational programme, delivered as a 62-minute video guided, facilitated discussion workshop:  Delivered as similar as possible on each of five experimental occasions  Pre-test post-test questionnaires undertaken  **Control group:**  Only completed the questionnaire. | Workshop consisted of:  (i) A video showing an elderly married couple discussing their sexuality, tasteful love making scenes, as well as open discussion of masturbation.  (ii) Followed by a 15-minute facilitated discussion by the presenter about the video.  (iii) A short video depicting the lifestyles of two married couples, who have partners with AD  (iv) Followed by facilitated discussion on sexual relationships where one partner has dementia and lives in an institution. | Pre-test n = 210 nursing staff:  (i) experimental group n = 120  (ii) control group n = 90  Post-test n = 183 nursing staff completed questionnaire:  (i) experimental group n = 101  (ii) control group n = 82  Participants worked in long-term care institutions. | **Attitude &**  **Knowledge**  Assessed via the Ageing and Sexuality Knowledge & Attitude Scale for Dementia (DEMASKAS).  **Demographic information:**  Age, gender, home language, education level & training. | Significant differences were found from pre to post-test on the three knowledge subscales of the DEMASKAS scale (F [1, 181] = 39.15; p = 0.001 in the pre to post-test x treatment group on the three knowledge subscales of the DEMASKAS scale (F [1, 181] = 19.08; p = 0.001 and in post-test scores were see in two of the subscales:  (i) knowledge on aging and sexuality - F [1, 181] = 6.36; p = 0.05  (ii) dementia and sexuality - F [1, 181] = 5.52 p = 0.05  **Repeated measures**  **MANOVAS:** significant differences were found from pre to post-test on the three attitude subscales:  (i) general morality (ii) institutional sexuality (iii) dementia & sexuality - *F* = 33.42; p = 0.001.  Analysis demonstrated a consistently more tolerant attitude towards alert patients than those with mild dementia. | Attitudes changed significantly in a more tolerant direction after an educational program, except towards patients with severe dementia.  Attitudes in the control group remained practically unaltered.  No significant correlation was seen between attitude change & demographic variables or prior knowledge of sexuality in dementia.  Demographic variables had little influence on attitude changes.  No significant changes were seen in the control group. | 24/42  57% |
| **Mayers & McBride (1998) [25]**  USA | Pilot study  To:  (i) educate staff regarding sexuality in aged care;  (ii) normalise the topic and address staff perceptions and prejudices;  (iii) offer information | Pilot training programme delivered as a x3 hour workshop  Delivered as group activities, open discussions and handouts to consolidate learning | Workshop focused on:  (i) Attitudes toward sexuality & the elderly;  (ii) Terminology & communication;  (iii) Residents' rights and abilities to make decisions about their sexuality;  (iv) Information sharing and handouts: information to carry away from the session & share with co-workers. | n = 27 including psychiatrists, non-psychiatrist physicians, nurses and nurse administrators, social workers, therapists & students.  ● 20 of the 27 participants undertook a post training interview | **Efficacy of the training:** assessed via a standard hospital feedback form, during workshop discussion  **Attitude &**  **Knowledge:** assessed via a brief sexual attitude survey & individualised interview five months post training | Only qualitative statements:  **(i) Workshop staff discussions.**  Participants valued discussion on:  (i) views perceptions of sexually attraction in older age.  (ii) how to discuss sexuality sensitively  (iii) developing a humanist approach to  resident sexuality  **(ii) Post training interviews:**  The majority of participants highly rated the workshop.  Post training interviews suggest:  (i) frequency of and ease of communication with staff increased  (ii) reduced embarrassment, discomfort or distress when talking to patients and other staff members about sexuality.  (iii) increased information  about older age sexuality, terminology, attitude and awareness and use in practice  Some participants reported that the written materials reinforced the validity of the topic, helped staff to understand the issues more clearly, clarified ideas & were helpful for future reference & documentation.  ***(iii) General attitudes about the workshop*** - All participants indicated that they liked the workshop. | The training program on sexuality was quite effective in eliciting interest & participation.  Methods used to enable staff to discuss the topic openly were well generally accepted. | 13/48  27% |
| **Menzel (2005) [26]**  USA | Quasi-experimental design.  Implementation of training program for staff to increase knowledge and awareness of sexuality in the elderly.  Three hypotheses:  (i) Experimental group will increase knowledge.  (ii) Experimental group will have positive change in attitude.  (iii) Experimental group will report fewer cases of ‘acting-out’ behaviours in the elderly pre and post-test.  No change in control group in knowledge, attitude or cases | Training programme consisting of x1 hour face-to-face presentation, supplemental handouts, video footage & in group discussion with case studies/case vignettes.  **Experimental group**: received training program. Pre & post-test measures were undertaken  **Control group** (a second nursing home): completed pre and post-test measures at the same time as the experimental group. | Programme had a three-part focus – knowledge, attitudes and practice.  **(i) Phase one:** focused on attitudes towards sexuality in the elderly.  **(ii) Phase two:** covered basic knowledge. This section included a video presentation featuring several vignettes of elderly couples discussing the effects that aging has on their sexuality; and professionals discussing their perceptions & experiences of sexuality in the elderly.  **(iii) Phase three**: an in-group discussion with case studies/case vignettes. Specific strategies were offered in response to the vignette examples. Focus was on problem solving & practical solutions. | n = 35 nursing home staff in western Pennsylvania:  (i) Experimental group n= 29 undertook training  (ii) Control group n = 6  n = 20 agreed to complete a questionnaire & take part in a short interview at the time of the pre- & post-test | **Knowledge -**assessed through researcher developed questionnaire.  **Attitude -**assessed through researcher developed questionnaire.  **Incidence of sexual behaviour in the past month** -assessed through interview questions. | Significant increase in the number of reported sexual behaviours from pre to post-test.  Significant differences were found in attitudes toward geriatric sexuality from pre to post-test.  Although no significant increase in knowledge of geriatric sexuality were found, the trend suggested that staff training had a positive impact. | Training programs could be beneficial, although this was not significant.  Trends in the data suggested support of the hypotheses  Significant decrease in reported incidence of sexual behaviour at post-test in experimental group; suggests that staff training had an impact on the reporting of these behaviours.  Assumed that the number of incidents had not decreased, but attitudes towards incidents had shifted.  The training programme had a greater impact than anticipated in relation to discussions about policy in nursing homes. | N/A - dissertation |
| **Reingold &**  **Burros (2004) [27]**  USA | Unclear  To explore the perceptions, challenges & opportunities of care staff in long-term care settings for the elderly. | A policy & video training program on sexual expression; duration not specified  Staff education was undertaken by a social worker & a psychiatric nurse clinician. | Care home policy & the importance of seeing sexual expression as right-based before a facilitated discussion around a range of  vignettes based upon actual cases.  Focus was placed on residents with dementia. | Focus on staff in one nursing home with seventeen units - nurses, aides,  physicians, social  workers, dieticians & activity co-ordinators. | None | Only qualitative statements were provided.  Reported that:  (i) staff education was received positively by staff  (ii) staff were relieved to have clearer guidelines on how to respond to situations which had made them anxious, uncertain, and uneasy. | Used vignettes in  educational  intervention.  No outcome measures utilised.  No specific  demographic  details reported. | 5/42  12% |
| **Steinke (1997) [28]**  USA | Pilot study  To assess the effects of an educational intervention on one sample of facility staff. | Education intervention on sexuality in ageing, delivered as two one-half day (one week apart) education sessions.  Participants completed a pre-test at the beginning of the first education session – demographic tool, ASKAS and three general questions related to sexuality & privacy issues.  Participants completed a post-test ASKAS at the end of the last half day session. | Educational methods included lecture, discussion, audio-visual aids, a game and printed materials.  **Programme content:**  (i) Perspectives on sexuality in ageing.  (ii) Assessment of sexual function.  (iii) Psychosocial concerns.  (iv) Approaches to sexuality in the nursing facility.  (v) Effects of chronic illness and medications on sexuality | n=10 (Female = 8  Male = 2) registered  nurses, licensed  practical nurses,  certified medication  assistants, certified  nursing assistants  **Age range:** 29-62; mean age = 39.2 years  **Years of experience:**  Range 1-20+years | **Attitude &**  **Knowledge:** assessed using ASKAS.  **Sexuality and privacy issues**: assessed through three general questions. | Significant increases seen in knowledge about sexuality from pre-test to post- test; t (9) = 4.27, p = 0.002.  No significant differences seen in  attitude scores from pre- to post-test; t (9) = 0.72, p = .49 | An educational intervention can increase nursing staff knowledge about sexuality in ageing.  There was no change in attitudes observed  intervention period; reported that staff had fairly permissive attitudes at the outset of the education programme & that attitudes usually occur over a longer period of time. | 15/42  36% |
| **Walker & Harrington (2002) [29]**  USA | Evaluation of a pilot study of four training modules/pre-test post-test design.  To:  (i) test the four modules with participants to ascertain the effectiveness & acceptability of the training prior to a planned field test and (ii) test the short-term effects of four training modules designed to improve staff knowledge & attitudes | Sex & Sexuality in Long Term Care curriculum, intended to be delivered over a three-week period.  Each session consisted of around an hour of instruction related to one of the four program topics.  Included long term care facilities did not offer the same modules, therefore duration varied.  Participants attended one or more training sessions  Each session included an introduction, videotape & a discussion of case studies.  Detailed instructions for presenting the session were provided in an instructor manual. | Project team identified 17 specific training objectives related to sexuality & the elderly that could serve as the blueprint for developing a training program.  Revised 17 objectives following a ranking study & focus group discussions were ordered into subtopics that became the basis for four modules  Instructor manual included: guidelines for presenting the materials, a transcript of the videotapes, step-by-step instructions for presenting the information, case study handouts, additional resources aimed at providing information to instructors & evaluation instruments (pre & post-tests, program evaluation forms).  Four main topics:  (i) The need for sexuality and intimacy; (ii) sexuality and dementia; (iii) sex and aging & (iv) family & personal issues. | Convenience sample of n = 109 (n= 99 female;  n= 10 male) long-term care staff (registered  nurses, licensed  practical nurses, nursing assistants, activity aides  and other professionals) from four settings who completed one or more of the four modules.  **Age range:** 20-69  (Mean = 38.47 years).  **Years of experience:**  1 to 33 (Mean = 9.14 years) | **Attitude &**  **Knowledge:**  Assessed using the Knowledge and Attitudes Toward Elderly Sexuality  (KATES) - developed by the team in previous research.  **Demographic information:**  Age, gender, ethnicity, marital status, education level, primary language, years’ experience. | **Training effect on knowledge & attitudes toward elderly sexuality:**  Main effect of module was not significant (p = .085) suggesting that across the pre- & post-tests participants performed at similar levels on all four modules.  Main effect of time (p< .0005) was significant, indicating that post-test scores were higher than pre-test scores.  Interaction of time by module (p< .0005) was significant, suggesting that the improvement from pre- to post-test was not uniform across the four modules.  Specifically, scores improved from pre- to post-test for three modules - need for sexuality and intimacy, sexuality and dementia, & sexuality & aging, but remained stable from pre to post-test for family & personal issues.  **Programme evaluation:**  n = 117 program evaluation forms.  89.8% of participants said that the information in the modules was useful or very useful.  89.7% said the information was very interesting or interesting respectively.  **Amount of information**: 81.2% said there was the right amount of detail.  **With regards to programme materials:** 91.4% felt that the handouts or visuals were useful or very useful & 94.9% thought videos they saw were very useful or useful. 92.3% said the case studies were very useful or useful. | Significant time effect suggests that the training modules were successful at improving long-term care staff knowledge of & attitudes toward elderly sexuality.  Significant time by  module interaction suggests that the improvement was not uniform across the four modules with only three areas: (i) need for sexuality & intimacy; (ii) sexuality & dementia; and  (iii) sexuality & aging showing increases of 57%, 24%, & 66% respectively.  Scores on the family & personal issues module stayed stable from pre to post-test - whist pre-test scores on this module were higher than for the other three modules, on average, participants getting 10 out of 15 items correct, suggesting that a ceiling effect is unlikely to account for the non-significant change from pre to post-test. | 32/42  76% |
| **White & Catania (1983) [30]**  USA | Randomized  control trial  To assess the effects of an older age-oriented sex education program on elderly persons, nursing home staff, and adult family members of elderly persons. | A sexual psychoeducational intervention developed to challenge the myths & create a permission giving climate regarding sexuality in old age among older persons, adult family members of older persons, and staff members of nursing homes.  **Duration:**  (i) Older adults: x3, 2-hour sessions  (ii) Family of older adults: 1-day, 6-hour session  (iii) Nursing home staff: 1-day, 6-hour session  All participant groups received same lecture material but the perspective altered depending on whether resident, staff or family member.  All experimental groups received pre & post-test measures via personal interview.  **Control group**: received pre & post-test measures via personal interview at the same time period as the experimental groups    Transcripts of each lecture were provided for the participants. | **Content/stimulus material:**  (i) Session l: lecture-discussion on the myths about sexuality and aging, followed by a panel discussion video tape of a group of experts on sexuality & aging;  (ii) Session 2: lecture-discussion on psychological and physiological aspects of sexuality and aging and a film on interpersonal intimacy;  (iii) Session 3: lecture on disease effects, drug effects, and environmental effects on sexuality and a simulation exercise on problem situations encountered by older persons regarding sexual activity. Lecture topics included material on the normative changes in sex & aging, benefits of sex in old age, social hindrances to sexual expression & intimacy, retirement, marriages & other physiological, pharmacological, psychological & sociological factors relevant to sex & aging.  The panel discussions video tape provided opinions and facts about sexuality & aging from professionals with backgrounds in religion, sexuality & aging. | **Nursing home staff participants:**  (i) Intervention N = 30  (ii) Controls = 33.1 years), i.e. administrators, registered and licensed practical nurses & activity directors, were recruited from local long-term care facilities.  **Older adults:**  n = 30 (12 men; 18 women) community residing elderly participants recruited from local Older American groups.  Mean age: Intervention =  67.8 years; Controls = 69.3 years)  Participants were all Caucasian & self-reported heterosexuals.  **Family of older adults:**  n = 30 family of elderly persons i.e. immediate relatives. | **(i) Attitude & Knowledge:**  Assessed using ASKAS scale  **(ii) Sexual behaviour questionnaire** for older adults only | Significant changes in knowledge about & attitudes towards sexuality & aging in all three participant experimental cohorts post intervention, exposure to programme had significant positive effect in all groups.  Intervention had no significant impact on sense of self-rated attractiveness.  Frequency of sexual fantasies did not change significantly post intervention.  Significant increase in recognising the importance of sexuality.  Significant increase (400%) in sexual behaviour in experimental group. | Significant changes in attitudes toward & knowledge about sexuality & aging & sexual behaviour. | 24/42  57% |
